# Lipocalin 2 as a potential systemic biomarker for central serous chorioretinopathy

**DOI:** 10.1038/s41598-020-77202-y

**Published:** 2020-11-19

**Authors:** A. Matet, T. Jaworski, E. Bousquet, J. Canonica, C. Gobeaux, A. Daruich, M. Zhao, M. Zola, M. Meester-Smoor, D. Mohabati, F. Jaisser, S. Yzer, F. Behar-Cohen

**Affiliations:** 1 Centre de Recherche Des Cordeliers Inserm, UMR_1138, Physiopathology of Ocular Diseaic Innovationsses/Therapeut, Université de Paris, Université Sorbonne Paris Cité, 15 rue de l’Ecole de Médecine, 75006 Paris, France; 2 Université de Paris, Paris, France; 3 Department of Ophthalmology, Institut Curie, Paris, France; 4 Ophthalmopole, Cochin University Hospital, Assistance Publique-Hôpitaux de Paris, Paris, France; 5 Jules-Gonin Eye Hospital, Department of Ophthalmology, University of Lausanne, Lausanne, Switzerland; 6 Service de Diagnostic Biologique Automatisé, Cochin Teaching Hospital, Paris, France; 7 Necker-Enfants Malades University Hospital, Department of Ophthalmology, Assistance Publique-Hôpitaux de Paris, Paris, France; 8 Department of Ophthalmology, Erasmus MC, Rotterdam, The Netherlands; 9 Rotterdam Ophthalmic Institute, Ophthalmic Institute, Rotterdam, The Netherlands; 10 Department of Ophthalmology, Leiden University Medical center, The Netherlands; 11 Rotterdam Eye Hospital, Rotterdam, The Netherlands

**Keywords:** Neuroscience, Molecular neuroscience, Oculomotor system, Visual system

## Abstract

No systemic biomarker of Central Serous Chorioretinopathy (CSCR) has been identified. Lipocalin 2 (LCN2 or NGAL), alone or complexed with MMP-9 (NGAL/MMP-9), is increased in several retinal disorders. Serum levels of LCN2 and NGAL/MMP-9 were measured in CSCR patients (n = 147) with chronic (n = 76) or acute/recurrent disease (n = 71) and in age- and sex-matched healthy controls (n = 130). Samples with CRP > 5 mg/L, creatinine > 100 µmol/L, and/or urea > 7.5 mmol/L were excluded. Serum LCN2 was lower in CSCR patients than controls (81.4 ± 48.7 vs 107.3 ± 44.5 ng/ml, p < 0.0001), and lower in acute/recurrent CSCR than controls (p < 0.001) and chronic CSCR (p = 0.006). Serum NGAL/MMP-9 was lower in CSCR patients than controls (47.2 ± 40.7 vs 74.1 ± 42.6, p < 0.0001), and lower in acute/recurrent CSCR than controls (p < 0.001) and chronic CSCR (p = 0.002). A ROC curve showed that for LCN2 serum levels, the 80-ng/ml cutoff value allows to discriminate acute/recurrent CSCR from controls with 80.3% sensitivity and 75.8% specificity, and for NGAL/MMP-9 serum levels, a 38-ng/ml cutoff value allows to discriminate acute/recurrent CSCR from controls with 69.6% sensitivity and 80.3% specificity. In both acute and chronic CSCR, low serum LCN2 and NGAL/MMP-9, provide a biological link between the two CSCR forms, and potential susceptibility to oxidative stress and innate immune dysregulation in CSCR.

## Introduction

Central serous chorioretinopathy (CSCR) is primarily an ocular disease, affecting the choroid and the retinal pigment epithelium (RPE). Different forms of the disease are recognized although a consensus nomenclature is still missing^1^. Most of the patients present with a focal RPE leak that causes a spontaneously resolving, and sometimes recurrent serous detachment. In a minority of patients, widespread retinal pigment epitheliopathy, and/or persistent serous detachments, result in severe visual impairment and anatomical complications^2^. The exact mechanisms and the causative factors for the disease remain uncertain. The most widely recognized risk factors for CSCR include exposure to exogenous or endogenous corticosteroids, psychopharmacologic medication use and type A personality, sleep disorders, shift work, helicobacter pylori infection, and cardiovascular risk factors such as hypertension and coronary heart disease^3^. Genetic predisposing factors have been identified, such as polymorphism in genes encoding complement factor system regulators^4^, the mineralocorticoid receptor^5^, plasminogen activators^6^ and VIP receptor^7^. For typical presentations the diagnosis of CSCR is usually straightforward, based on clinical imaging features, such as spectral domain optical coherence tomography (SD-OCT), fluorescein angiography (FA) and indocyanine green (ICG) angiography. However, it can be challenging in atypical cases, particularly in women, subjects with thin choroid on SD-OCT or those without favoring factors. To the best of our knowledge, no systemic biomarker of CSCR has been identified to date.

Lipocalin-2 (LCN2), whose human ortholog is Neutrophil Gelatinase-Associated Lipocalin (NGAL), is a 25-kD secreted protein, expressed in numerous cell types and organ systems, such as innate immune cells, epithelial cells, and brain astrocytes. In the eye, it is expressed in retinal pigment epithelial cells^8^ and in retinal glial Müller cells ^9^. LCN2 is induced in response to acute injury, infection and metabolic disturbance by NF-κB activation^10,11^. However, depending on the disease phase (acute vs chronic) and on the injured organ, LCN2 exerts pro- or anti-inflammatory effects. In transgenic mice with defective lysosome-mediated clearance in RPE cells, harboring phenotypic features of early age-related macular degeneration, LCN2 produced by neutrophils enhances their retinal infiltration, contributing to age-related changes^12^. On the other hand, in brain^13^ and eye models of lipopolysaccharide-induced inflammation, LCN2 reduced inflammation through the inactivation of NF-κB^9^. An additional function of LCN2 is to promote matrix metallopeptidase 9 (MMP-9) activity by forming a complex with the protease (NGAL/MMP-9)^14^.

In the retina, the role of LCN2 is incompletely understood. It is the most expressed early-stress gene in the RPE and the neurosensory retina of *Abca4*^*-/-*^* Rdh8*^*-/-*^ mice overexposed to light, a model of AMD. In this model, LCN2 protected from gliosis and microglial activation, as shown in *Lcn2*^-/-^ *Abca4*^-/-^ *Rdh8*^-/-^ mice^8,15^. In addition, LCN2 protected human RPE cells derived from induced pluripotent stem cells (iPS-RPE cells) against oxidative stress by increasing the expression of the antioxidant enzymes HMOX1 (Heme oxygenase 1) and SOD2 (superoxide dismutase 2)^15^, suggesting that LCN2 exerts important neuroprotective effects in the outer retina.

LCN2 is employed as a biomarker for several inflammatory and metabolic diseases^16^, and is recognized as one of the best diagnostic and prognostic marker for acute kidney injury^17–21^. LCN2 is also a biomarker for atherosclerosis, myocardial infarction and heart failure^22^. After myocardial infarction, LCN2 produced by neutrophils induces the polarization of macrophages towards a phenotype that allows clearance of apoptotic cells and reduces cardiac fibrosis. LCN2 is thus beneficial for cardiac remodeling ^23^.

In ocular diseases, systemic and local LCN2 levels have also been measured. In central retinal vein occlusion, LCN2 is increased in aqueous humor but not in serum^24^. In diabetic retinopathy, plasma LCN2 levels are elevated and correlate with the severity of retinopathy^25^. In AMD, plasma LCN2 levels are elevated and LCN2 is increased in aqueous humor of patients with wet AMD^26^. To our knowledge, no study has measured the systemic levels of LCN2 or NGAL/MMP-9 in CSCR. The primary aim of this study was to investigate the potential of LCN2 and NGAL/MMP-9 as biomarkers for CSCR. Therefore, we compared serum LCN2 levels in subjects with different forms of CSCR, and in healthy controls.

## Subjects and methods

Patients were included from 3 cohorts recruited at the Jules-Gonin Eye Hospital (Lausanne, Switzerland), the Ophthalmopole Cochin hospital (Paris, France) and the Rotterdam Eye Hospital (Rotterdam, Netherland). The cases selection was based on the availability of samples from patients with well-defined phenotypes based on multimodal retinal imaging.

**Figure 1:**
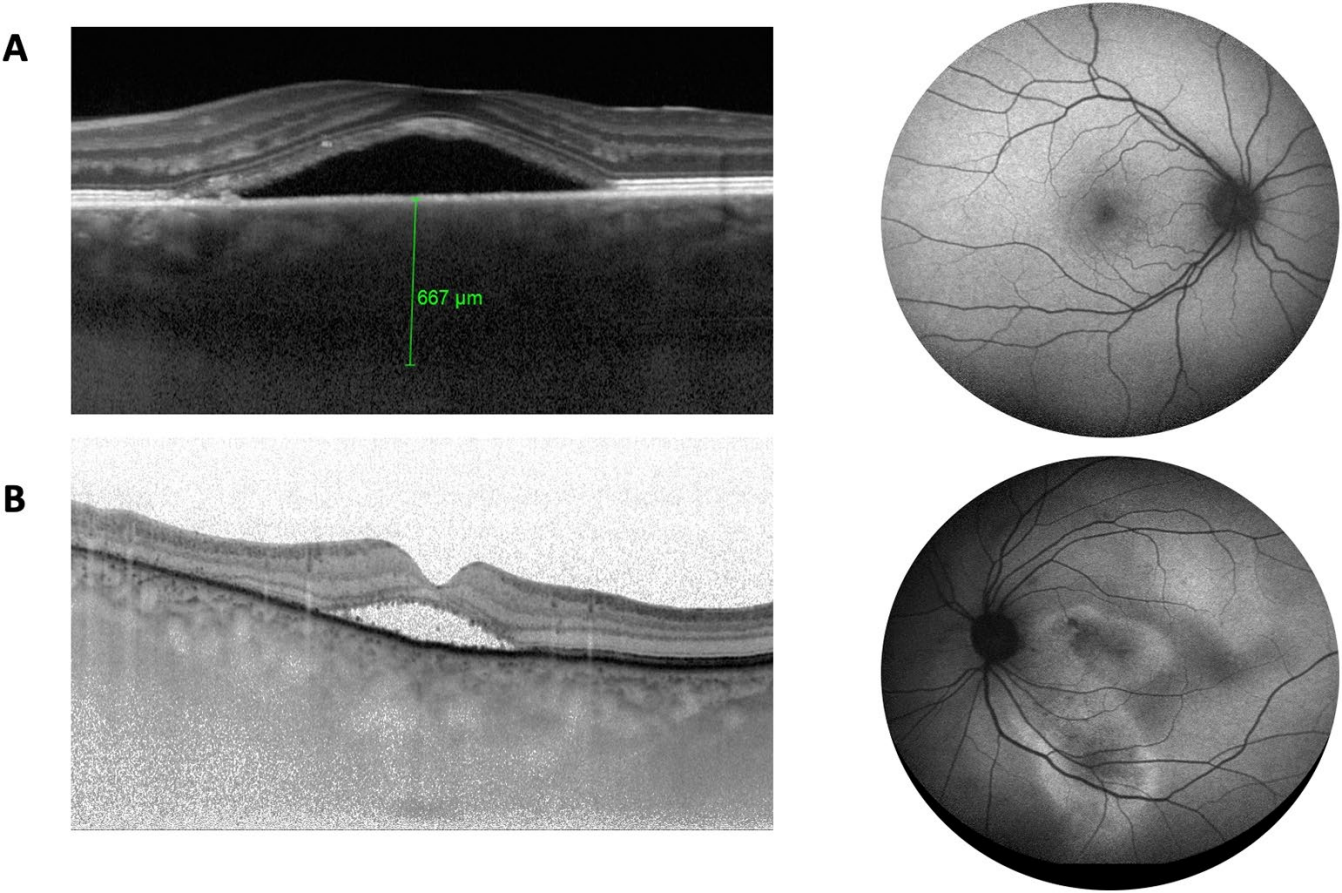
Representative horizontal cross sections of the retina on an enhanced depth imaging spectral domain OCT in an acute central serous chorioretinopathy (CSCR) (**A**), and a chronic CSCR (**B**) patient. On the blue autofluorescence imaging there is limited retinal pigment epithelium (RPE) alteration (< 2 disc diameters) in the acute CSCR (**A**), while in the chronic CSCR (**B**), an extended area of pigment epithelium alteration is observed.

The study is a cohort case–control retrospective study. Diagnostic criteria for CSCR were defined on multimodal imaging including FAF, spectral-domain optical coherence tomography (SD-OCT, Spectralis, Heidelberg Engineering, Heidelberg, Germany) and FA. Patients were divided in two groups based on the disease history and/or the presence of an underlying multifocal epitheliopathy as observed on FAF and on FA. Patients with epitheliopathy (total cumulated area > 2 disc diameters) were classified as chronic cases, while patients without epitheliopathy were classified as acute/recurrent cases (Fig. 1). ICG angiography was performed in all patients in Paris and Lausanne clinics and only in patients with epitheliopathy to exclude choroidal neovascularization or for treatment guidance according to the decision of the attending clinician. Patients with any other ocular disease, such as age-related macular degeneration (characterized by the presence of drusens), diabetic retinopathy, retinal vein occlusion, high myopia > -6D, or glaucoma were excluded from the study. Since OCT-A was not available in all clinics at the time of cohorts constitution, choroidal neovascularization (CNV) secondary to CSCR could not be excluded with certainty, although patients suspected of CNV on SD-OCT were excluded if CNV was confirmed on ICG.

Serum samples were withdrawn at different hours during the day depending on clinic organization since previous study did not found any circadian influence on systemic LCL2 levels^27^. All patients had an active episode of CSCR at the time of blood sampling defined as the presence of subretinal fluid. Serum samples from control subjects were obtained from the Banque Française du Sang (BFS) under an agreement between BFS and Inserm. Blood samples were collected from donors aged between 20 and 60 years, preferentially men who had no previous history of ocular diseases to match with the age and sex ratio of the CSCR patients, although no significant correlations between systemic LCN2 levels and age or sex have been observed in other cohorts^28,29^.

### Ethics statement

This research was conducted in compliance with the tenets of the Declaration of Helsinki and was approved by the competent institutional review boards in France (Comité de Protection des Personnes CPP Ile de France 1, protocole C16-09 N°DC-2016–2620), Switzerland (La Commission cantonale (VD) d'éthique de la recherche sur l'être humain (CER -VD) Eyeomics340/15) and in the Netherlands (Medische Ethische Toetsingscommissie Leids Universitair Medisch Centrum, NL50816.058.14). Written informed consent was obtained from each patient and healthy participant.

### LCN2 and NGAL/MMP-9 serum level measurements

Human Lipocalin-2/NGAL Quantikine ELISA Kit and Human MMP-9/NGAL Complex Quantikine ELISA Kit (R &D Systems, catalog number DLCN20 and DM9L20 respectively, Minneapolis, MN) were used to measure NGAL and NGAL/MMP-9 complex according to the manufacturer protocol. All samples were tested in duplicated and required a 20-Fold dilution.

Because LCN2 levels are influenced by kidney function^21^ and by inflammatory state^16^, patients with CRP > 5 mg/L, creatinine > 100 µmol/L, urea > 7.5 mmol/L were excluded (53 and 24 subjects were excluded in the CSCR and control groups, respectively). ELISA analyses were performed in Paris in 2019 by JC and TJ blind to diagnoses.

### Statistics

Descriptive, comparative and correlative statistics were computed on XLstat software (version 2014.6.01; Addinsoft, Paris, France). Quantitative values were expressed as mean ± standard deviation. Categorical variables were presented as counts and percentage. The Kolmogorov–Smirnov test was employed to assess the normal or non-normal distribution of quantitative values. The Mann–Whitney test or the Kruskal–Wallis test with post hoc Dunn test were employed to compare quantitative values. The Chi-square test was employed to compare proportions. Receiver operating characteristics (ROC) curves were plotted and analyzed to assess the sensitivity, specificity, and cutoff values of serum marker levels. The optimal cutoff was found using the Youden index. P values inferior to 0.05 were considered significant.

## Results

### Demographic characteristics

The demographic characteristics of 147 patients with CSCR and 130 healthy control subjects are shown in Table 1. Mean age and sex ratio did not differ statistically between the CSCR and control groups. In the CSCR group, 71 patients (48%) presented an acute or recurrent form of the disease, while 76 patients (52%) presented a chronic form of the disease with epitheliopathy.

**Table 1:** Data of patients with central serous chorioretinopathy (CSCR) and healthy controls included in the study.

Group	CSCR patients (n = 147)	Healthy controls (n = 130)	p value
Age, mean ± SD, years	48.7 ± 8.6	47.2 ± 10.6	0.4^a^
Sex ratio, male, n (%)	120 (81.6%)	103 (79.2%)	0.6^b^
NGAL serum level (ng/ml)	81 ± 48.7	107.3 ± 44.5	** < 0.0001**^**a**^
NGAL/MMP9 serum level (ng/ml)	47.2 ± 40.7	74.1 ± 42.6	** < 0.0001**^**a**^

^a^Mann-Whitney U test; ^b^Khi2 test.

NGAL: Neutrophil Gelatinase-Associated Lipocalin.

MMP-9: Matrix metallopeptidase 9. Bold p values indicate different groups

### Serum levels of LCN2 (NGAL) and NGAL/MMP-9 are lower in CSCR than in control subjects

**Figure 2:**
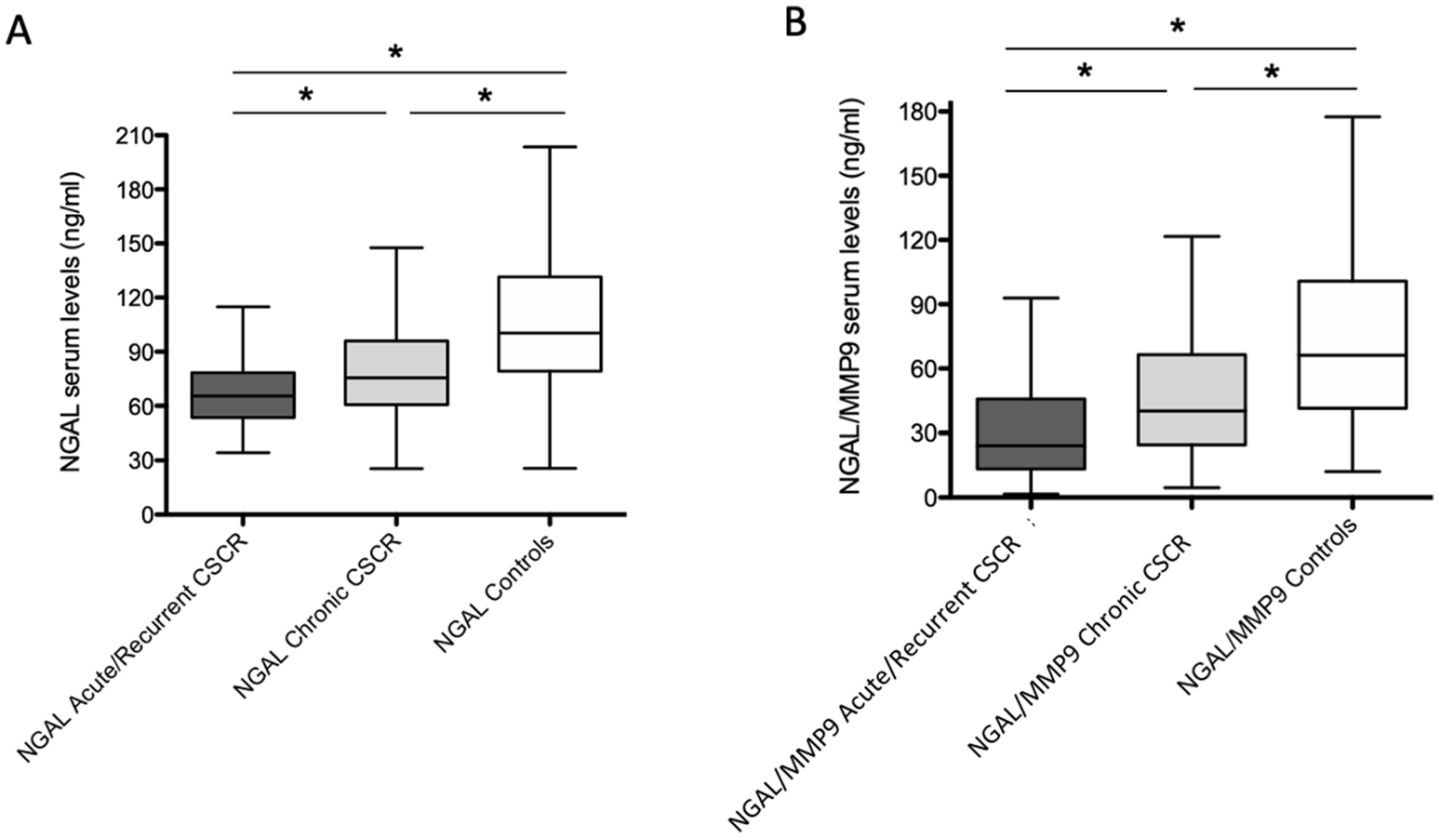
Serum NGAL (**A**) and NGAL/MMP-9 (**B**) levels (ng/ml). Mean ± SD serum levels in CSCR as compared to control subjects. ***p < 0.001, **p < 0.01; NGAL: Neutrophil Gelatinase-Associated Lipocalin; MMP-9: Matrix metallopeptidase 9.

Serum levels of LCN2 (NGAL) and the LCN2/MMP-9 (NGAL/MMP-9) complex are shown in Table 1, Table 2 and in Fig. 2. Serum NGAL was significantly lower in the CSCR cohort than in the control group (81 ± 48.7 ng/ml vs 107.3 ± 44.5 ng/ml respectively, p < 0.0001, Table 1). In patients with CSCR, serum NGAL level was significantly lower in the acute/recurrent subgroup than in the chronic subgroup (p = 0.006, Table 2). In both forms of CSCR, NGAL level was lower than in the control group (p < 0.001, Table 2).

**Table 2:** Serum levels of NGAL and NGAL/MMP9 in patients with acute/recurrent CSCR, chronic CSCR and healthy controls.

	Acute/recurrent CSCR^a^ (n = 71)	Chronic CSCR^b^ (n = 76)	Healthy controls^c^ (n = 130)	p value*
NGAL serum level (ng/ml)	71.6 ± 33.4	89.9 ± 58.6	107.3 ± 44.5	^a^ vs ^b^ : p = 0.006
				^a^ vs ^c^ : p < 0.001
				^b^ vs ^c^ : p < 0.001
NGAL/MMP9 serum level (ng /ml)	38.9 ± 38.4	54.8 ± 41.5	74.1 ± 42.6	^a^ vs ^b^ : p = 0.002
				^a^ vs ^c^ : p < 0.001
				^b^ vs ^c^ : p < 0.001

*Kruskal–Wallis test with post hoc Dunn test and Bonferroni correction.

CSCR: central serous chorioretinopathy.

NGAL: Neutrophil Gelatinase-Associated Lipocalin.

MMP-9: Matrix metallopeptidase 9.

Similarly, serum NGAL/MMP-9 levels were significantly lower in CSCR patients as compared to controls (47.2 ± 40.7 ng/ml vs 74.1 ± 42.6 ng/ml, respectively, p < 0.0001, Table 1). Serum NGAL/MMP-9 level was significantly lower in the acute/recurrent CSCR subgroup than in the chronic CSCR subgroup (p = 0.002, Table 2). In both forms of CSCR, NGAL/MMP-9 level was lower than in the control group (p < 0.001, Table 2).

### ROC curve analysis

**Figure 3:**
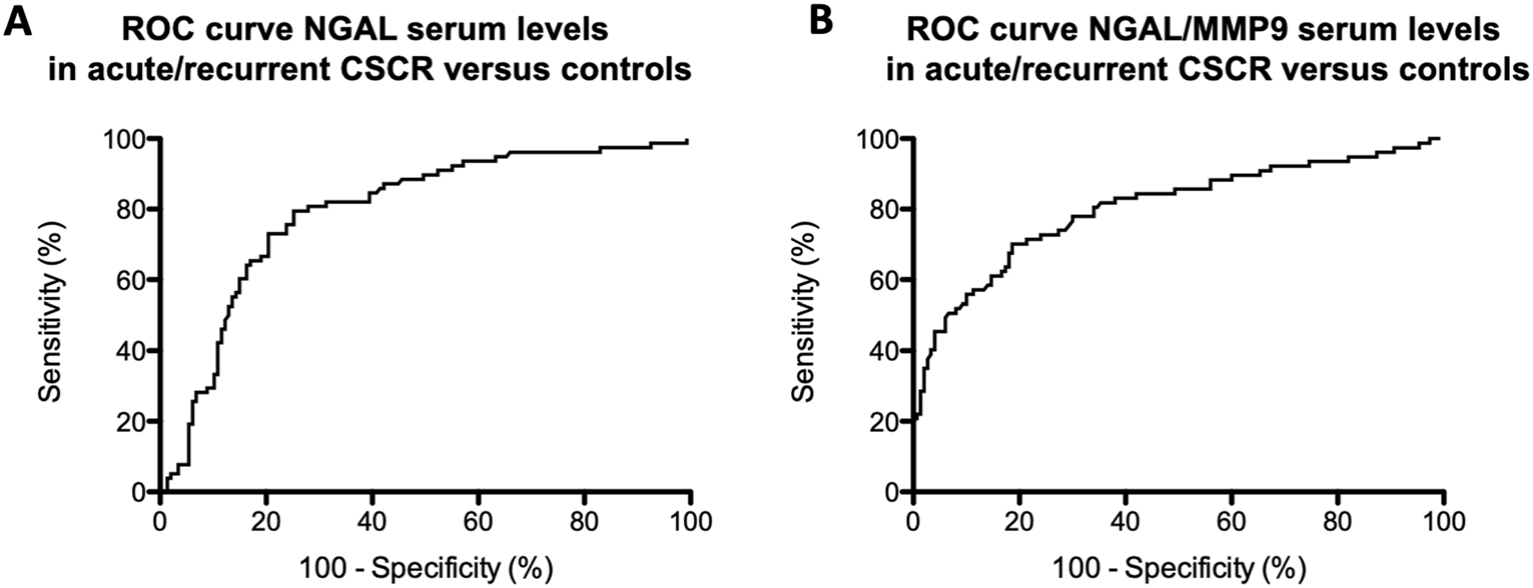
Receiver operating characteristics (ROC) curves for NGAL (**A**) and NGAL/MMP-9 (**B**). AUC: Area Under the Curve. NGAL: Neutrophil Gelatinase-Associated Lipocalin; MMP-9: Matrix metallopeptidase 9.

As displayed in Fig. 3, a ROC curve analysis showed that for serum levels of LCN2 (NGAL), an optimal cutoff value (Youden index) of 80 ng/mL allowed to discriminate acute/recurrent CSCR (< 80 ng/mL) from controls (≥ 80 ng/mL) with 80.3% sensitivity and 75.8% specificity. For serum levels of the NGAL/MMP-9 complex, the optimal cutoff value (Youden index) of 38 ng/mL allowed to discriminate acute/recurrent CSCR (< 38 ng/mL) from controls (≥ 38 ng/mL) with 69.6% sensitivity and 80.3% specificity.

## Discussion

In this study, we observed that patients with CSCR have lower serum levels of LCN2 (NGAL) and NGAL/MMP-9 than control subjects. The fact that patients originate from 3 different cohorts reinforce the strength of this unexpected finding. Serum levels measured in control subjects from the present study are in the range measured in control populations of two previous studies (115 ± 86 ng/ml, n = 30^30^, and 122.53 ± 26.15 ng/ml, n = 142^31^). High systemic level of LCN2 is a biomarker of metabolic^16^, cardiac ^32^, and acute kidney disease^18,19^. Plasma LCN2 has been identified both as diagnostic and severity marker of diabetic retinopathy^25^. Increased plasma LCN2 levels were also measured in patients with Stargardt disease, retinitis pigmentosa, and AMD, as compared to healthy controls^8^. Yet, our findings surprisingly indicate that in CSCR patients LCN2 serum levels are lower compared to healthy controls. In addition, levels of the NGAL/MMP-9 complex are also lower, suggesting a decreased endogenous production of LCN2 in CSCR patients. Moreover, according to our subgroup observations, subjects with retinal pigment epitheliopathy (chronic CSCR), and those without RPE damage (acute/recurrent CSCR) have reduced LCN2 and NGAL/MMP-9 serum levels. These findings suggest a biological continuum between both forms of the disease and indicate a potential underlying mechanism. As compared to other organ systems, where LCN2 exerts rather pro-inflammatory effects, LCN2 showed anti-inflammatory and anti-oxidant effects in the retina, and particularly in models of pre-existing RPE pathology, such as in *Abca4*^*-/-*^*Rdh8*^*-/-*^ mice submitted to light^15^. LCN2 was also shown to enhance the proper membrane distribution of ZO-1 and VE-Cadherin^33^, which could be important because in CSCR, RPE junctions could be disrupted. Recently, Parmar et al.showed that LCN2 exerted a strong dose-dependent protective effect on human iPS-RPE against H_2_O_2_-induced cell death, and from inflammatory-induced apoptosis. In addition, the expression of the LCN2 receptor *SLC22A17* gene was upregulated by light in RPE cells, showing that LCN2, either produced by RPE cells or by immune cells, may serve to protect the retina from inflammation and oxidative stress-induced degeneration^8^. Reduced serum LCN2 levels in CSCR patients could thus confer excessive susceptibility to oxidative stress and contribute to the alteration of the RPE barrier function. This is supported by the recent observation that the disulfide/thiol ratio is significantly greater in CSCR patients than in healthy control subjects^34^.

On the other hand, low levels of the NGAL/MMP-9 complex may protect the retina from MMP-9 activity and subsequent leukocyte infiltration. Indeed, in the brain, MMP-9 is required for the initial infiltration of leukocytes through the blood–brain barrier in experimental autoimmune encephalomyelitis^35^. Leukocyte infiltration in the retina has been observed in patients with AMD as a consequence of the AKT2-NFkB-LCN2 axis^12^. In CSCR, low MMP-9 activity could contribute to the maintenance of the inner blood-retinal barrier, despite outer retinal barrier breakdown.

Another interesting observation is that LCN2 is one of the rare NF-kB-induced molecules^10^ that is up-regulated by glucocorticoids^36^. The paradoxical predisposing and aggravating effects of glucocorticoids in CSCR, could result from an improper regulation of LCN2 by glucocorticoids. But, whether serum levels of LCN2 and NGAL/MMP-9 reflect their ocular levels, and whether these levels vary differently upon corticoid simulation in CSCR patients as compared to healthy controls, remain to be studied.

There are some weaknesses in this study and particularly the absence of ICG angiography and OCT-A for all patients included in the different clinics, due to different practice and to the fact that OCT-A was not available in all clinics when patients were included. Thus, we cannot exclude with certainty that some chronic patients did not have CNV, or a very atypical form of AMD, since OCT-A was not systematically performed in all the patients. Yet, the clinical diagnosis of chronic CSCR also relies on clinical history and risk factors, which differ from AMD patients. Systemic LCN2 being increased in patients with wet AMD^8^, this could in part explain why complex CSCR with epitheliopathy in our study had higher LCN2 levels than acute cases. Whether LCN2 could help discriminate CSCR patients with CNV is currently under study. In this study, we have not investigated correlations between functional clinical parameters, such as visual acuity, and LCN2, LCN2/MMP9 levels, which will be the subject of an ongoing prospective study.

In conclusion, the observation of decreased serum levels of NGAL (LCN2) and NGAL/MMP-9 in CSCR patients with and without retinal pigment epitheliopathy, provides a biologic link between the two forms of the disease and, suggests a potential mechanism consistent with the current understanding of the disease pathogenesis. To our knowledge, CSCR is the only ocular disease associated with decreased LCN2 level, and therefore LCN2 could be potentially used as a biomarker for CSCR, particularly in atypical cases or those where the differential diagnosis with AMD is challenging. Further comparative studies should be conducted to confirm this preliminary finding.
